# EHMTI-0384. A double blind randomized placebo controlled trial for non-invasive dynamic trans-cutaneous electrical nerves stimulation in management of tension type headaches

**DOI:** 10.1186/1129-2377-15-S1-J2

**Published:** 2014-09-18

**Authors:** L Akhmadeeva, D Valeeva, M Naprienko, L Goldobina, G Rayanova, B Veytsman

**Affiliations:** 1 Neurology, Bashkortostan State Medical University, Ufa, Russia; 2 Pediatrics, Bashkortostan State Medical University, Ufa, Russia; 3 Non-pharmacological Methods of Care, First Moscow State Medical Univeristy, Moscow, Russia; 4 Physical Therapy @ University Hospital, Bashkortostan State Medical University, Ufa, Russia; 5 Neurology @ University Hospital, Bashkortostan State Medical University, Ufa, Russia; 6 Computational Sciences, George Mason University, Fairfax, USA

## 

Tension-type headaches (TTH) are the most common type of headaches, and new non-invasive modalities might improve their better management. Dynamic transcutaneous electrical nerves stimulation (dTENS) is one of them. We designed a protocol for a randomized double blind placebo controlled clinical trial to assess the effects of dTENS for patients with chronic TTH. dTENS is added to the standard management with no changes in routine practice. We invite for this study adolescents, adults and elderly patients who consented to participate. Specially designed headache diaries, HALT, HART indices and clinical interview are included as the major outcomes measures. The zones, intensity and duration for dTENS application were recommended and modified by physical therapist (L.G.) and acupuncture specialist (M.N.). They included the zones (on the head and neck) and dosage recommended by the manufacturer. The course of dTENS study consists of 10 procedures performed by clinicians (D.V. and G.R.) who were trained for this. All participants of the study are randomized into two groups using a computer-based software. Placebo-devices were made for this trial by the manufacturer (DENAS Corporation, Ekaterinburg, Russia). The sham-devices look and sound exactly as the active ones, but electrodes are not connected to the electrical stimulator. These compact devices for dTENS are approved for using in medical settings and at home, and this trial might add evidence for physicians when prescribing them to patients.
